# Ovarian Cancer: The Fallopian Tube as the Site of Origin and Opportunities for Prevention

**DOI:** 10.3389/fonc.2016.00108

**Published:** 2016-05-02

**Authors:** Sophia H. L. George, Ruslan Garcia, Brian M. Slomovitz

**Affiliations:** ^1^Department of Obstetrics and Gynecology, Division of Gynecology Oncology, Miller School of Medicine, University of Miami, Miami, FL, USA; ^2^Sylvester Comprehensive Cancer Center, Miller School of Medicine, University of Miami, Miami, FL, USA

**Keywords:** salpingectomy, BRCA1, BRCA2, ovarian cancers, fallopian tubes

## Abstract

High-grade serous carcinoma (HGSC) is the most common and aggressive histotype of epithelial ovarian cancer (EOC), and it is the predominant histotype associated with hereditary breast and ovarian cancer syndrome (HBOC). Mutations in *BRCA1* and *BRCA2* are responsible for most of the known causes of HBOC, while mutations in mismatch repair genes and several genes of moderate penetrance are responsible for the remaining known hereditary risk. Women with a history of familial ovarian cancer or with known germline mutations in highly penetrant genes are offered the option of risk-reducing surgery that involves the removal of the ovaries and fallopian tubes (salpingo-oophorectomy). Growing evidence now supports the fallopian tube epithelia as an etiological site for the development of HGSC and consequently, salpingectomy alone is emerging as a prophylactic option. This review discusses the site of origin of EOC, the rationale for risk-reducing salpingectomy in the high-risk population, and opportunities for salpingectomy in the low-risk population.

## Introduction

In 2015, approximately 22,000 women in the United States were diagnosed with ovarian cancer and 14,000 died from this devastating disease (1). Ovarian cancer is a heterogeneous disease that can be divided into three main types: sex cord stromal tumors, germ cell tumors, and epithelial ovarian cancer (EOC). EOC accounts for the vast majority of ovarian cancers and consists of different subtypes, namely, mucinous, endometrioid, clear cell, low-grade serous, and high-grade serous carcinoma (HGSC) (2). The various histotypes differ in epidemiology, etiology, and treatment. High-grade serous ovarian carcinoma is not only the most common subtype of EOC, accounting for 75% of cases, but also the most aggressive. Most women present at advanced stages (stage III or IV) at diagnosis, at which point the 5-year survival rate ranges between 20 and 40%. However, for patients with stage I disease, the 5-year survival rate exceeds 90% (3). Molecular and genetic data indicate that HGSC of the ovary may have a similar origin to HGSC of the fallopian tube and peritoneum, and therefore, it has been suggested that all the three be described collectively as HGSC (4). Of the patients diagnosed with a HGSC, 15–20% will have a known germline mutation in the highly penetrant homologous repair pathway genes, *BRCA1* or *BRCA2*.

In the general population, the incidence of ovarian cancer is higher in white women than in women from other racial or ethnic groups, and survival rates at 12 years are better in Caucasian American women (38%) compared with African-American women (32%). Of interest, Hispanic women (43%) and Asian women (52%) have higher survival rates. It is estimated that about 1 in 500 Americans have a mutation in *BRCA1* or *BRCA2*. The lifetime risk of developing ovarian cancer with germline mutations in *BRCA1* and *BRCA2* is 40–60 and 11–27%, respectively (5–9). The burden of hereditary breast and ovarian cancer syndrome (HBOC) was previously thought to be confined to white women, particularly those of Ashkenazi Jewish descent. However, recent studies of different immigrant populations in the United States and in their respective countries of origin have identified pockets of women who bare a similarly high genetic burden as the Ashkenazi Jewish population. Women of Bahamian heritage, for example, are estimated to have 27.1% of breast cancer cases due to *BRCA* mutations (10, 11). The ovarian cancer burden in these isolated high-risk populations is still unclear, but likely to be as high as those women of Ashkenazi descent. Other highly penetrant genes, such as *PTEN* and *TP53*, and moderately penetrant genes, such as *PALB2*, *BRIP1*, *CHEK2*, and *ATM* (12), are also associated with HBOC, albeit at lower frequencies than the prevalence of *BRCA1* and *BRCA2* mutations. Norquist et al. recently reported *RAD51C*, *RAD51D*, and *BARD1* as additional genes mutated in the germline of invasive serous ovarian cancer patients (12, 13). These data suggest that despite the growing list of genes involved in ovarian cancer predisposition, 70–85% of the women diagnosed with HGSC have “sporadic” disease.

Ovarian cancer incidence and mortality among US women has declined in those aged 35–59 years due to earlier detection methods or changes in risk (3). Conversely, in Southern and Eastern Europe, there is a corresponding rise in incidence (14) as women reduce breastfeeding and have fewer children (decrease in parity), which are both known risk factors. A similar trend of increasing incidence is expected in low–middle income countries (14).

Screening methods with CA-125 and transvaginal ultrasound have proved mostly ineffective in decreasing mortality for sporadic HGSC and ovarian cancer in general (15, 16). Early detection has been and continues to be a challenge in ovarian cancer because the disease is habitually asymptomatic before peritoneal spread (17). However, with the identification of pockets of the population at high risk for HBOC, there is an opportunity to reduce the burden of disease through increased and targeted genetic testing as well as screening and prevention measures for ovarian cancer risk reduction.

## Cell of Origin of Serous Ovarian Cancer

### Ovarian Surface Epithelia

Prior to the reported observation of *in situ* carcinoma in the distal end of the fallopian tube of women undergoing prophylactic surgery, the ovary was thought to be the etiological site of high-grade serous ovarian cancer. Now, there are two candidates for the cell of origin, namely, the fallopian tube epithelium (FTE) and the ovarian surface epithelium (OSE). Both share common mesodermal embryological origin and close anatomic proximity. The fallopian tube, along with the uterus, uterine endocervix, and superior aspect of the vagina are derived from an invagination of the celom known as the Mullerian or paramesonephric ducts. The OSE is derived from the mesothelial celomic epithelium that lines the primitive ovary (18).

The “incessant ovulation” hypothesis, proposed by Fathalla (19), suggested that continuous ovulatory cycles during the reproductive lifespan of a woman increase her risk of developing HGSC (19). He proposed that ovulation resulted in an increase in inflammation through which the secretion of cytokines, chemokines, bradykines, and hormones induce DNA damage *via* oxidative stress in the cortical inclusion cysts (CIC) observed in the ovary. These events, along with proliferation of the OSE, promote metaplastic changes leading to neoplastic transformation (2, 15, 19).

Xenografts of transformed OSE cell lines and genetic animal models have been used in an attempt to model HGSC in the absence of *in situ* pre-neoplastic lesions in the ovary. Genetic mouse models deleting *BRCA1*, *Rb1*, and *TP53* genes from the OSE resulted in leiomyosarcomas (20) and not HGSC. In contrast, targeted deletion of these genes in the fallopian tube epithelia of mice has led to the development of tumors genomically and pathologically similar to HGSC (21). The somatic mutational spectrum found in lesions associated with the ovary proper and neoplastic lesions have been shown to have *KRAS*, *BRAF*, *CTNNB1*, *ARID1A*, *PTEN, PPP2R1A*, and *PIK3CA* (22). These tumors rarely have *TP53* mutations, which suggest a distinct etiology and natural history of tumorigenesis from that of HGSC.

### Fallopian Tube Epithelia

There is now substantial convincing clinical and molecular evidence in support of the FTE as the source of the cell of origin of low- and high-grade serous ovarian cancer (22). Experimental *in vitro* manipulation and transformation of human fallopian tube epithelial cells have demonstrated that these cells in a xenograft model can give rise to tumors, which resemble primary HGSC (23). Additionally, mouse models targeting *BRCA* and *TP53* in fallopian tube epithelia develop HGSC (21).

A series of transcriptional studies by Tone et al. and George et al. have shown that the phenotypically normal fallopian tube epithelia from *BRCA1* and *BRCA2* mutation carriers show transcriptional differences when compared to epithelial cells with a normal *BRCA* genotype. These differences have been shown to impact different molecular pathways. Consequently, these pathways are implicated in tumor initiation, progression, and recurrence (24–26). As a result of these studies, the authors proposed that chronic inflammatory states through cyclical ovulation in the presence of a mutated *BRCA* allele could predispose the normal FTE to undergo neoplastic transformation, which may lead to serous carcinoma. This would primarily occur through deregulation of DNA damage response genes and synergistically through upregulation of cytokines, proinflammatory and proliferation genes.

The *BRCA*-associated carcinomas share some common genomic features such as frequent mutations of *TP53* and copy number landscape features including *Cyclin-E1* amplification and deletion of *Rb1* (27). Altered BRCA function is not unique to hereditary HGSC but is prevalent *via* somatic mutations (6%) (28–31), promoter hypermethylation (13–31%) (28, 32–34), and other genetic or epigenetic alterations, predominantly in the homologous recombination (HR) pathway in HGSC. This has led to determining the “BRCAness” profile in patients (35, 36). Overall, these differences in morphologically normal epithelia from *BRCA* mutation carriers have shed light into the effects of heterozygosity and predisposition to the development of HGSC and, importantly, potential features that might be manifested in the STIC.

Detailed histopathological examination of tubal epithelia in *BRCA* mutation carriers undergoing risk-reducing surgery led to the discovery of putative cancer precursor lesions in the fallopian tube referred to as serous tubal intraepithelial carcinoma (STIC) (37–40). STIC was first reported by Piek et al., who described dysplastic epithelial changes in the fallopian tubes of women with a *BRCA1* or *BRCA2* mutation, who underwent risk-reducing salpingo-oophorectomies (RRSO) (38, 41). These lesions have distinct morphological features such as loss of polarity, epithelial tufting, and pleomorphic nuclei, and in addition, there is abnormal p53 expression and a high-proliferative index (refer to Lheureux et al. for commentary) (2, 42).

Since the discovery of the STIC, three possible pre-neoplastic lesions have been described, including the p53-signature, low grade serous tubal intraepithelial lesions (STIL), and secretory cell outgrowths (SCOUTS) (43). These lesions share a combination of phenotypic and/or genomic alterations with the cancer cells in HGSC. *TP53* mutations, which are ubiquitous in HGSC, are usually concomitantly found in STIC and HGSC (2, 44). Over-expression of p16 has been documented in some STILS and over-expression of Pax8, Bcl-2, and loss of Pax2 expression has been observed in SCOUTS (45). However, none of these lesions are clinically actionable, as it is still unclear which of these lesions and/or combination of genomic alterations, has the pathogenic capacity to give rise to a carcinoma.

Many studies have now reported the incidence of non-invasive neoplastic lesions (STIC) in the distal end of the fallopian tube. It is estimated that occult invasive and STIC are identified in 0.9–8.5% of women undergoing RRSOs (2, 39, 40, 43, 46–63) (Table 1). The frequency of STIC lesions increases with age and is lower with oral contraceptive use (64). It is important to note that the large range in estimates of the prevalence of occult and STIC lesions is reflective of the variances in diagnostic methodologies used by different centers and study groups (42).

**Table 1:** **Evidence of serous tubal intraepithelial carcinoma in risk-reducing salpingo-oophorectomy of asymptomatic women with known *BRCA1* or *BRCA2* mutations or strong family history of breast or ovarian cancer**.

Reference	Number of RRSO cases	Incidence of STIC or occult carcinoma in the distal end of the fallopian tube
Colgan et al. (53)	60	5 (8.3%)
Piek et al. (41)	12	5 (41.6%)
Leeper et al. (55)	30	3 (10%)
Powell et al. (49)	67	4 (6%)
Carcangiu et al. (56)	50	4 (8%)
Finch, et al. (48)	159	7 (4.4%)
Callahan et al. (52)	122	7 (5.7%)
Shaw et al. (39)	176	15 (8%)
Hirst et al. (54)	45	4 (8.9%)
Powell et al. (59)	111	6 (5.4%)
Manchanda et al. (50)	117	10 (8.5%)
Mingels et al. (58)	226	16 (7.1%)
Reitsma et al. (60)	303	3 (0.99%)
Wethington et al. (62)	593	12 (2.0%)
Cass et al. (57)	78	9 (11.5%)
Sherman et al. (61)	966	25 (2.6%)

Powell and colleagues reported that in a long-term follow-up study of women diagnosed with non-invasive serous tubal epithelial carcinoma who underwent RRSO, 6% (1/17) recurred 43 months after risk-reducing surgery compared to 43% of women who had unsuspected invasive carcinoma at time of surgery (65). There is a continued need to understand the effects of inflammation and hormones on the fallopian tube epithelia, relating to latency and the preferential sites of seeding, are critical for addressing prevention and risk-reduction strategies in genetically high-risk populations.

### Opportunities for Ovarian Cancer Risk Reduction

Epidemiological data show that oral contraceptives, aspirin, and other non-steroidal anti-inflammatory drugs reduce the risk of ovarian cancer. In a meta-analysis as a primary prevention mechanism by Havrilesky et al., oral contraceptive pills use reduced ovarian cancer risk by 50% if used for more than 10 years (66). Recently, aspirin use was associated with a reduced risk of ovarian cancer, especially among daily users of low-dose aspirin (67). These observations highlight the relationship between ovulation and its inflammatory accompaniment with ovarian cancer development. Women identified at highest risk, that is, germline mutation carriers and/or strong family history of ovarian cancer, may benefit from use of these chemoprevention strategies.

Tubal ligation (tubal sterilization) has been shown to reduce ovarian cancer risk that theoretically is spread through retrograde menstrual flow (68–70). In particular, tubal ligation was associated with reduced risk of invasive ovarian cancer, with the greatest benefit seen in the endometrioid and clear cell subtypes (71). The mechanism of protection is through prevention of retrograde menstruation, and hence, a decrease in Fenton’s reaction (generates reactive oxidative species) in the environment of the fallopian tube as well as prevention of endometrial cells implanting in the ovary. Although tubal ligation appears to be protective for all histotypes of ovarian cancer, it is least effective in reducing risk for the most lethal subtype, HGSC (71).

### Salpingectomy

As early as 2002, Rebbeck et al. suggested that bilateral prophylactic oophorectomies reduced the risk of ovarian and breast cancer in women with *BRCA1* or *BRCA2* mutations by as much as 96% (72). Olivier et al. demonstrated that risk-reducing salpingo-oophorectomy reduced the risk of ovarian, fallopian tube, and peritoneal papillary serous carcinoma in *BRCA1* and *BRCA2* mutation carriers (some women still developed peritoneal disease) (73).

**Figure 1:**
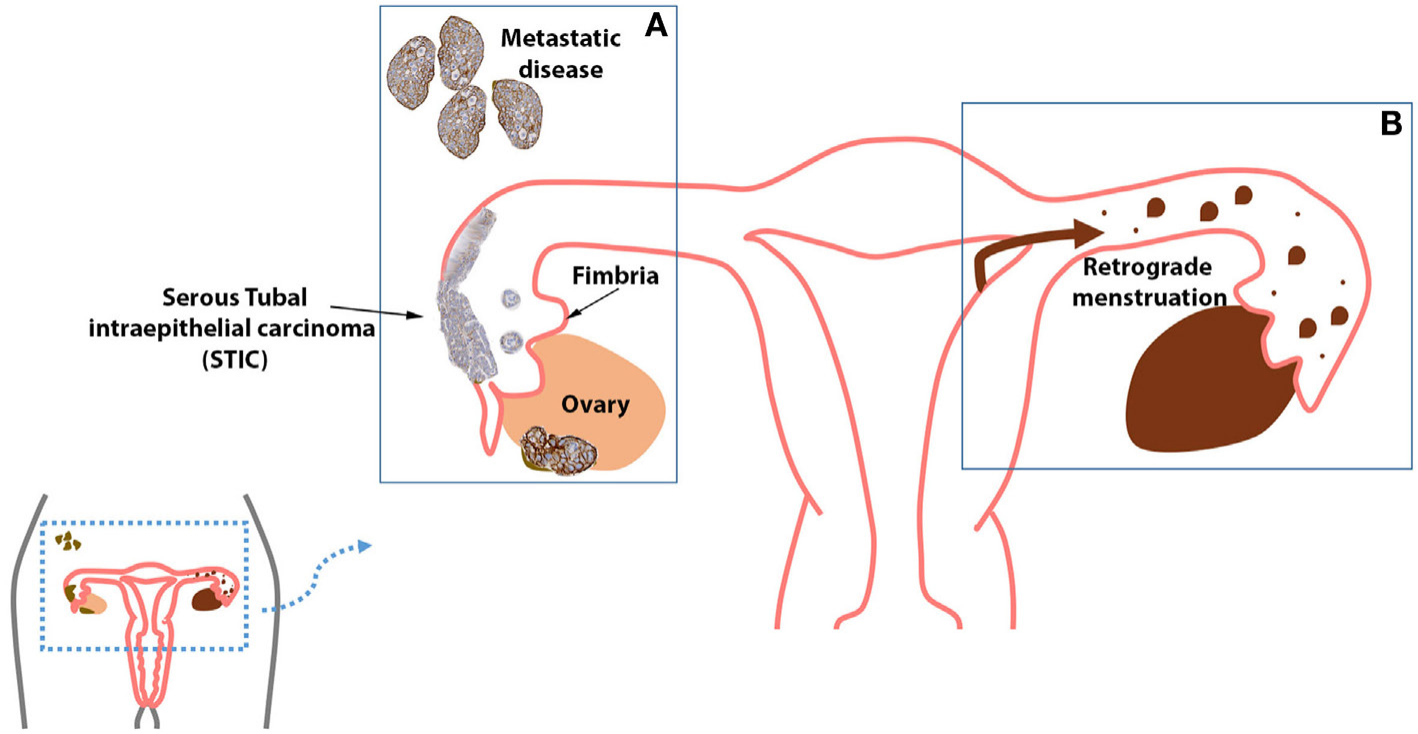
**(A)** High-grade serous carcinoma (HGSC) is the most common histologic type of cancer seen in the ovaries, fallopian tube, and peritoneum. **(B)** The fallopian tubes may act as a possible conduit for retrograde menstrual flow, which is theorized to induce the malignant transformation of cells *via* oxidative stress, inflammation, and hyperestrogenism. Further, endometriosis has been strongly linked with endometrioid ovarian carcinoma and clear cell ovarian carcinoma.

As previously mentioned, there is clear evidence supporting the role of the fallopian tube as the etiological site of HGSC [and most likely low-grade serous carcinoma (22)]. For this reason, women with known risk for breast and ovarian cancer may undergo prophylactic surgical removal of the ovaries and fallopian tubes, a procedure known as RRSO. Current guidelines from the National Comprehensive Cancer Network and the Society of Gynecologic Oncologists suggest that RRSO to be completed by the post-child bearing period, the age of 35–40, or 10 years younger than a first-degree relative diagnosed with ovarian cancer (74). However, it is believed that the majority of these high-risk women do not undergo RRSO by age 40 (75). This modality of precision prevention involves risk stratification and risk reduction in patients carrying both highly penetrant (76) (*BRCA1* and *BRCA2*) and moderate to lower penetrant genes such as *PTEN*, *PALB2*, *CHEK2*, *ATM*, and *BRIP1*. The removal of the fallopian tubes alone is referred to as risk-reducing salpingectomy (RRS). In young women identified with a *BRCA1* or *BRCA2* mutation, RRS is performed in an effort to reduce ovarian cancer risk while maintaining adequate hormonal levels to avoid the effects of early menopause. This latter approach, however, is not restricted to women at high risk for serous ovarian cancer, as it will also have a beneficial impact on reducing the risk of development of endometriosis-associated clear cell and endometrioid ovarian cancer (Figure 1).

### Opportunistic Salpingectomy

Opportunistic salpingectomy refers to removal of the fallopian tubes in women who are *not* at an increased risk of developing ovarian cancer. In 2010, a population-based and institution-wide study in British Columbia, Canada, was initiated whereby three recommendations to gynecological surgeons were made: (1) consider opportunistic salpingectomy during hysterectomy, (2) consider excisional bilateral salpingectomy rather than tubal ligation for sterilization, and (3) refer all HGSC patients for *BRCA1/2* germline testing (77). Interim results on surgical outcomes revealed that the rates of hysterectomy with bilateral salpingectomy increased 3.5-fold compared to hysterectomy alone, and the rates of tubal ligation as a mode of surgical sterility decreased from 99.7% in 2009 to 66.7% in 2011, while the rate of bilateral salpingectomy concomitantly increased 111-fold compared to 2009 rates (77). The authors also reported that the length of hospitalization post-hysterectomy and bilateral salpingectomy was not longer than for hysterectomy alone and that there was no significant difference in the rate of blood transfusion or hospital readmission among these two groups. In addition, there was no significant difference in length of hospitalization or rate of transfusion for bilateral salpingectomy compared to tubal ligation.

The caveat to opportunistic salpingectomy is that even if implemented on a large scale, the true impact of ovarian cancer reduction will take years to be realized (77). It is also important to note that salpingectomy alone, unlike oophorectomy, does not reduce the risk of breast cancer by modulating levels of estrogen.

There is categorical evidence that RRSO reduces ovarian and breast cancer death and all-cause mortality (78, 79). There is currently no evidence that points to the outcome and impact of ovarian cancer risk reduction for two-stage procedure of salpingectomy followed by oophorectomy. In the United States, MD Anderson is conducting a clinical trial assessing prophylactic salpingectomy with delayed oophorectomy (80). A report from the Nurses’ Health Study concluded that compared with ovarian conservation, bilateral oophorectomy at the time of hysterectomy for benign disease was associated with a decreased risk of breast and ovarian cancer but an increased risk of all-cause mortality (81, 82); therefore, one can stipulate that salpingectomy alone may be sufficient in the genetically “low-risk” population, while the overall benefit versus harm of these approaches requires close attention in the genetically high-risk population. Specifically, oophorectomy offers protection against breast cancer even after menopause and improves survival in those with breast cancer (83). As these prevention modalities are implemented, it is important that the goal of decreasing the incidence and burden of ovarian cancer is not at the expense of worsening the incidence and mortality of breast cancer in women who are at increased risk due to co-morbidities.

## Author Contributions

SG wrote and conceptualized. RG researched references and made figure. BS wrote and conceptualized.

## Conflict of Interest Statement

The authors declare that the research was conducted in the absence of any commercial or financial relationships that could be construed as a potential conflict of interest.
